# Supplementary material for: Proteomic and Metabolomic Profiles of T Cell-Derived Exosomes Isolated from Human Plasma
Source: Cells. 2022 Jun 18;11(12):1965. doi: 10.3390/cells11121965 (PMC9222142; doi:10.3390/cells11121965)
Supplement: Supplementary file 1 [file Supplementary Figure S1.pdf › Supplementary Files/Supplementary Figure S1.pdf]

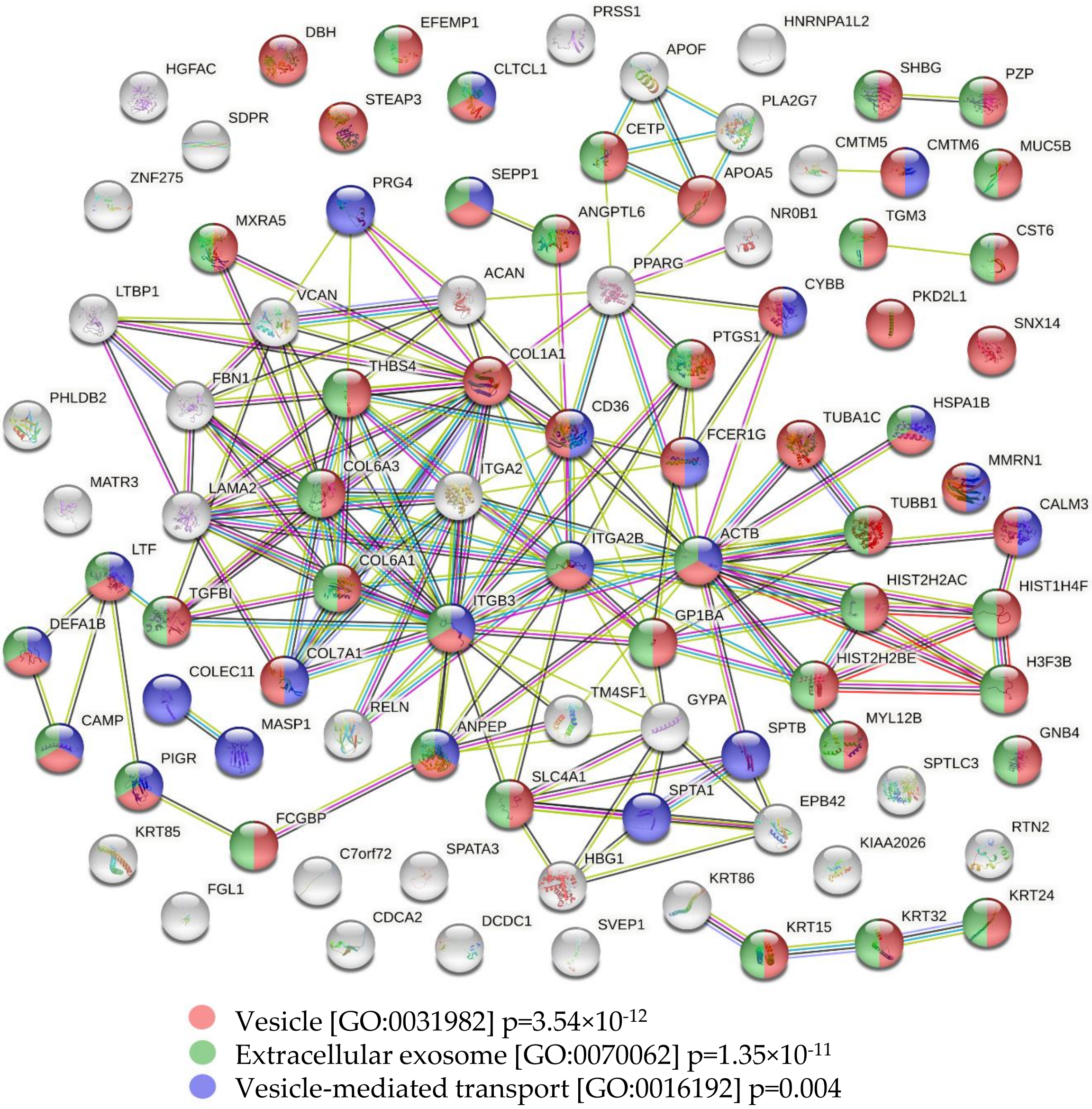

Figure S1: The functional network of proteins characteristic for the CD3(+)exo fraction that were not detected in CD3+/CD4+/CD8- T cells. Proteins that were associated with the selected biological processes are color-coded, along with the significance of the process overrepresentation. The putative interactions between the proteins and associated processes were found using the STRINGdb database.
